# A mixed methods study on continuity and care coordination based on the obstetric near miss approach

**DOI:** 10.4102/hsag.v29i0.2421

**Published:** 2024-04-22

**Authors:** Samuel M. Mulongo, Doreen Kaura, Bob Mash

**Affiliations:** 1 Department of Nursing, Faculty of Medicine and Health Sciences, Stellenbosch University, Cape Town, South Africa; 2 Department of Family and Emergency Medicine, Stellenbosch University, Cape Town, South Africa

**Keywords:** longitudinal continuity, relational continuity, parallel coordination, sequential coordination, access, obstetric near misses

## Abstract

**Background:**

The near-miss approach assumes that mothers facing life-threatening conditions such as severe pre-eclampsia and postpartum haemorrhage share common risk factors. Among these women, those who survive (near-miss cases) can offer insights into the determinants, providing valuable lessons for understanding underlying factors.

**Aim:**

To investigate elements of continuity and coordination leading to obstetric near misses.

**Setting:**

A major referral hospital and its referral pathway in Kenya.

**Methods:**

Explanatory sequential mixed-methods design.

**Results:**

Near-miss survivors had lower continuity and coordination of care indices during antenatal visits (COCI = 0.80, *p* = 0.0026), (modified continuity of care index [MCCI] = 0.62, *p* = 0.034), and those with non-life-threatening morbidity in the first trimester were more likely to experience a near miss (aOR = 4.34, *p* = 0.001). Facilities in the western region had a higher burden of near misses compared to the Eastern region. Qualitatively, three deductive themes were identified: *sequential coordination, parallel coordination and continuity*, along with factors classified as *access.* In mixed integration, poor continuity indices were explained by quality of interpersonal relationships and woman centredness. Poor coordination was explained by inadequate teamwork between providers in referring and referral facilities and between primary health facilities and the community. Higher near-miss rates in the western region resulted from differences in human and physical resources.

**Conclusion:**

Promoting woman-centred care, teamwork, improving communication and introducing innovative coordination roles like case and care managers can enhance continuity and coordination of maternal healthcare.

**Contributions:**

This study contributes to our understanding of the challenges of continuity and coordination in maternal healthcare in resource-poor settings by applying the WHO operationalisation of continuity and coordination using mixed methodology.

## Introduction

The high number of near-death experiences during pregnancy and childbirth poses a significant public health issue, disproportionately affecting socially and economically disadvantaged women (Goldenberg et al. 2017; Mekango et al. 2017). Severe maternal illnesses can leave lasting negative effects on a woman’s physical, mental and social well-being, impacting her ability to engage in social and economic activities (Storeng et al. 2010). To prevent near misses and ultimately reduce mortality, person-centred care emphasising *continuity* and *coordination* between patients and healthcare providers is crucial.

Given the decline in maternal deaths within facilities, adopting the near-miss approach, rather than the mortality approach, is essential for evaluating the quality of care. Gaining insights from near-miss survivors to understand failures in continuity and coordination in care pathways is vital, as it complements the mortality approach. Therefore, incorporating the experiences and perspectives of women and healthcare providers through qualitative approaches (in addition to epidemiological approaches) is critical (Donetto et al. 2015). This is especially critical in Kenya, where the decentralisation of healthcare created many layers of health services, highlighting the need for continuity to ensure effective coordination. While increased resources for counties resulted in notable infrastructure improvements, research suggests that positive maternal outcomes are not guaranteed without changes in human and organisational behaviour (Souza et al. 2013). In addition, the devolution of health services demands enhanced coordination within and between service points because of the expanded layers of health facilities.

This study aimed to utilise the World Health Organization’s operationalisation of continuity and coordination of care (Table 1) to examine the challenges faced by near-miss survivors in accessing healthcare in Kenya.

**TABLE 1:** Dimensions of continuity and coordination.

Construct	Definition
Longitudinal continuity	The degree to which a client attends the same provider over time
Relational continuity	Entails the quality of the relationship between a client and their provider.
Sequential coordination	Collaboration across facilities or levels of care
Parallel coordination	Collaboration within facilities or the same level of care

*Source:* WHO Secretariat, 2016, *Continuity and coordination of care a practice brief to support implementation of the WHO framework on integrated people-centred health services*, viewed 10 January 2020, from https://apps.who.int/iris/bitstream/handle/10665/274628/9789241514033-eng.pdf?ua=1

**TABLE 2:** Near miss criteria for retrospective near miss case identification.

Disease-specific criteria	Management-specific criteria	Organ dysfunction criteria
Severe pre-eclampsia, eclampsia, severe postpartum haemorrhage, ruptured uterus, sepsis or severe systemic infection, severe complication of abortion	Blood transfusion, emergency hysterectomy, interventional radiology	Cardiovascular, respiratory, renal, coagulation and/or haematological, hepatic, neurological or uterine dysfunction

*Source:* World Health Organization, 2011, ‘The WHO near-miss approach for maternal health’, *Bulletin of the World Health Organization* 87(10), 29. https://doi.org/10.2471/BLT.09.071001

### Theoretical underpinning

This study was grounded on Activity Development and Analysis (ACTAD) framework, a socio-technical theory that considers multiple factors, both internal and external, in shaping and improving practices (Anja et al. 2007). The ACTAD framework posits that human and organisational activity is defined by its object or objective, with mental capacities, community or context, tools, rules and division of labour as additional essential elements. In this study, the ACTAD framework was employed to identify coordination and continuity processes that contribute to obstetric near misses and to investigate how *tensions* and *conflicts* in the continuum of care can compromise care coordination and handling of obstetric emergencies. By examining the interconnectedness of women’s experiences and care activities, including *contradictions* that occur throughout pregnancy, childbirth and postpartum, activity theory offers insights into how *collective actors, means of communication and individual actions* influence maternal outcomes. Overall, activity theory provides a comprehensive framework for understanding and improving the coordination and continuity of care in obstetric settings.

### Literature review

Sandall et al. conducted a systematic review on the effect of longitudinal and relational continuity, which involves a pregnant woman consulting the same midwife throughout pregnancy. This approach was associated with a range of positive outcomes, including lower chances of regional analgesia, instrumental deliveries, preterm birth, foetal loss after 24 weeks and episiotomy (Sandall et al. 2016). Other studies have also shown that longitudinal and relational continuity enhances early presentation to the antenatal clinic for a subsequent pregnancy (Jinga et al. 2019; Nattey et al. 2018), reduces suicidal ideation (Duggan & Adejumo 2012; Knettel et al. 2020) and enhances general satisfaction with care (Sandall et al. 2016). *Group antenatal care,* where women of the same gestation attend their appointment as a group rather than individually is associated with more satisfaction but not improved perinatal outcomes (Catling et al. 2015). Therefore, literature establishes a strong link between continuity of care and satisfaction and other intermediate outcomes but lacks clarity on its association with severe morbidity, which is essential for policy decisions. Current frameworks inadequately detail activities influencing continuity and coordination, particularly in the causal pathway leading to maternal near misses (MNCs).

## Purpose

### Rationale

The existing near-miss literature does not fully capture the complexity of obstetric near miss and fails to incorporate the perspectives of near-miss survivors. Mixed-methods research can help bridge the gaps in understanding the factors contributing to severe maternal morbidity and death by offering a comprehensive and nuanced perspective on the coordination and continuity processes that affect near obstetric misses. In particular, qualitative research complements statistical approaches by providing a deeper understanding of the personal and circumstantial factors that contribute to severe maternal morbidity and death. Moreover, the WHO near-miss framework proposes the qualitative approach as a baseline assessment for near-miss survivors, nurses, doctors and other health system actors (World Health Organization 2011), emphasising the importance of including multiple perspectives in obstetric near-miss studies.

### Objectives

***Quantitative:*** (1) To investigate determinants of obstetric near misses in the catchment population of a major teaching and referral hospital *in this country* and (2) to compare continuity and coordination of care among near-miss survivors and mothers with normal deliveries.

***Qualitative:*** (1) To explore the perspectives of near-miss survivors and healthcare professionals on continuity and coordination of care and (2) observe activities that lead to obstetric near misses in primary healthcare facilities.

***Mixed:*** To investigate how quantitative findings obtained through retrospective chart reviews and cross-sectional survey are explained by subsequent qualitative results gathered from interviews and focus group discussions.

### Information sources and weighting

For the quantitative phase of the research study, the key sources of information were a census of all deliveries that took place in the hospital between 2019 and 2020, as well as survey data gathered from post-natal mothers. For the qualitative strand, the data sources included healthcare providers and purposively selected near-miss survivors from two designated facilities. The overall approach adopted was *building or development*, with equal weight given to each research arm. Building refers to the intention of using the data collection and analysis of one type of data to inform the sampling and data collection approach of the other type.

## Research methods and design

### Study design

**FIGURE 1:**
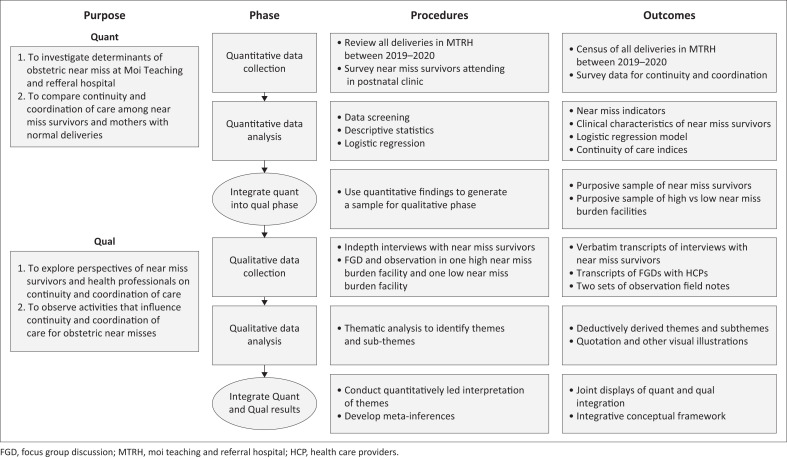
Procedural diagram for mixed-methods study on continuity and coordination based on obstetric near-miss approach.

This article is the fourth in a series of articles evaluating continuity and care coordination based on the obstetric near-miss approach. The study was a *two-phase, explanatory, sequential, mixed-methods design*. Findings from the quantitative phase informed sampling and development of the qualitative strand’s interview guides for data collection. Integration of qualitative and quantitative results enabled a deeper understanding of continuity and coordination elements that lead to the occurrence of obstetric near misses within and between health facilities. The overall study design is summarised in Figure 1.

***Quantitative:*** This phase used two nested designs: a retrospective chart review to identify near-miss cases and a cross-sectional survey to compare self-reported continuity and coordination of care. The researcher retrieved obstetric charts of 9823 mothers who sought care at Moi Teaching and Referral Hospital between 2019 and 2020 and surveyed 211 mothers who attended the post-natal clinic in May 2020. Of these, 99 were near-miss survivors, while 112 were normal deliveries. The WHO near-miss criteria were used to identify potential near-miss cases, and a Likert scale was used to elicit information on continuity and coordination of care. The study used descriptive analysis, multivariable logistic regression and Mann–Whitney U tests to analyse the data and determine the association between variables.

***Qualitative:*** This study was an exploratory, descriptive qualitative case study that targeted near-miss survivors and healthcare providers in the catchment population of the *hospital under study*. Participants were purposively selected based on the prevalence of morbidities underlying near miss and determinants that were statistically significant in the quantitative phase and facilities with the highest and lowest near miss burden. The study used three data collection tools: an individual interview guide, a Focus Group Discussion (FGD) guide and observation journal. The analysis and interpretation of data followed the generic steps for thematic analysis of qualitative data.

### Data collection process

***Quantitative:*** For the first objective, the authors retrieved 9823 obstetric charts of mothers who sought care for pregnancy, delivery or puerperium, between 2019 and 2020 at the referral hospital from sentinel units (maternity units, ICU and post-natal wards). The authors then used the WHO near-miss criteria (Table 1) to identify all potential near-miss cases. To address the second objective, the authors surveyed 211 mothers who attended the post-natal clinic in May 2020. Of these, 99 were near-miss survivors, while 112 were normal deliveries.

***Qualitative:*** Near-miss survivors, health providers and health facilities were all purposively selected based on the results of the quantitative phase. The authors used the following criteria: (1) prevalence of morbidities underlying near miss, (2) determinants that the authors found to be statistically significant in the multivariable analysis, (3) facilities with the highest and lowest near-miss burden. Based on these criteria, the following categories were selected: (1) nine survivors of severe postpartum haemorrhage and six survivors of eclampsia, (2) participants who attended tier four (primary referral facilities) and (3) one facility located on the western and one on the eastern side of the referral hospital.

### Data collection instruments

***Quantitative:*** For objective 1, the authors used the WHO near-miss data abstraction tool to collect data on the following variables: (1) maternal and perinatal information, (2) facility process indicators, (3) underlying causes of near miss and (4) contributory or associated conditions. In addition, the authors collected data on socio-demographic, clinical and obstetric factors for near-miss cases and normal pregnancies to enable comparison. To compare self-reported continuity and coordination of care between near misses and normal delivery cases (objective 2), the authors used a five-point Likert scale adapted from the Nijmegen continuity questionnaire (Uijen et al. 2012). The tool was designed to elicit information on the number and sequence of antenatal visits, the type of providers seen, the name of the facility for each visit and self-reported continuity and coordination of care. The authors modified the wording to reflect antenatal consultation and added items on sequential care coordination.

***Qualitative:*** The authors developed three data collection tools to gain insight into the perspectives of women and healthcare workers regarding determinants of obstetric near-miss cases. These tools included an individual interview guide, a FGD guide and observation journal. The principal researcher piloted the individual interview and FGD guides before administering them in the field. In addition, the observation checklist described the workflow processes that enabled interpersonal longitudinal and informational continuity, as well as the barriers and enhancers of sequential and parallel care coordination. The principal researcher (SM) collected all data between May and July 2021. The observed elements provided insight into the differences between areas with low versus those with high near-miss cases.

### Data processing

***Quantitative:*** The principal investigator aimed to collect high-quality data in a retrospective chart review by implementing various measures. These included using standardised electronic data abstraction forms for uniformity and easy access, developing a manual of standard operating procedures (SOPs) in line with good clinical practice and training research assistants in coding and abstraction techniques. Data handling involved a systematic process, beginning with data cleaning to identify and rectify any inconsistencies, errors or missing values. Next, data were entered into SPSS version 26. This system also facilitated efficient data storage and retrieval, contributing to the overall quality and reliability of the collected data.

***Qualitative:*** All key informant interviews, FGD guides and observation field notes were recorded and transcribed verbatim. Atlas Ti analysis software was used to assist with data analysis.

### Data analysis

***Quantitative:*** For objective 1, the authors used descriptive analysis to calculate: (1) maternal near-miss ratio (i.e. near miss cases per 1000 live births), (2) neonatal mortality and stillbirth rates, (3) prevalence of morbidities for disease-specific, management specific and organ dysfunction criteria and (4) prevalence of both direct and indirect causes of near miss for the three criteria. The authors used multivariable logistic regression to determine the association of socio-demographic and clinical variables with the occurrence of a near miss. For objective 2, the authors calculated two indices that measure the density and dispersion of antenatal visits (Jee & Cabana 2006). They include: (1) *Continuity of care* (*COC*) *index,* which measures the dispersion of visits by assigning a higher value to women who visit the same antenatal clinic and (2) *the Modified continuity index* (*MCI*), which is adjusted for utilisation by assigning a higher value to those with more frequent visits to the same providers. For all indices, a value of 1.0 was considered perfect longitudinal continuity, 0.75–0.99 was considered high, 0.50–0.74 medium and below 0.50 was regarded as poor continuity (Dery, Aikins & Maya 2020). The authors then compared these indices for near miss versus non-near miss cases using Mann–Whitney U tests.

The Likert scale was analysed as a multidimensional scale using means, standard deviations and Mann–Whitney U test. *Continuity of care* indices were entered into a binary logistic regression model to test their effect on near-miss occurrence, adjusting for socio-demographic and antenatal characteristics.

***Qualitative:*** The analysis and interpretation of data followed the generic steps for thematic analysis of qualitative data (Yin 2018). Transcribed data were first read line by line to determine the general perception of participants regarding the near-miss phenomenon and care processes. Common threads, words and phrases were noticed. Data coding was performed by searching the transcripts for statements that reflect unique opinions and experiences. The authors then used these to develop emerging patterns. The researcher used memos to document his thoughts regarding the data extensively. Because of the large number of codes generated, similar codes were merged before creating categories. Finally, codes were grouped into categories and themes were deductively developed from the categories.

### Data synthesis and integration

Integration during analysis aimed to elicit how qualitative findings help to understand the quantitative results. Mixed analysis followed an independent intra-method approach, where quantitative and qualitative data were analysed separately (as described above). The authors inferred meaning within each analysis and compared the findings to make interpretations. Firstly, the authors generated a mixed data collection inventory to visualise all data sources from the two phases. As many data points as possible were included in the list. Secondly, mixed-methods descriptive analysis was conducted to describe patterns in the data. The authors identified data linkages using a back-and-forth exchange. The authors then organised linked data using joint displays and checked it for complementarity and divergence. To handle divergence, the authors reverted to theory to find explanations and examine intra-method findings for potential biases.

### Rigour

***Quantitative:*** All data collection instruments were examined for validity and reliability indices. Reliability indices for the WHO near miss tools were reported based on results from previous validation studies. For instruments developed by the researcher, reliability indices were established during the pre-test stage of the research. Data integrity was ensured by anonymising all questionnaires and handling all collected data using the principles of good clinical practice in patient-oriented research. Methods, tools and findings were peer reviewed by supervisors and experts in the field.

***Qualitative:*** Credibility was established through conducting member checks and engaging in peer debriefing. The accuracy of the information was further ensured by confirming observations, conducting brief interviews and utilising software analysis. Dependability was attained by thoroughly documenting the procedures and decisions made during the research process, enabling others to assess their reputability and reasonableness. Confirmability was maintained by focusing on accurately representing participants’ experiences and ideas, rather than imposing the researcher’s own perceptions. This was accomplished through participant training, precise data transfer and preserving an audit trail throughout the research process.

### Ethical considerations

Ethical clearance to conduct this study was obtained from the Stellenbosch University, Health Research Ethics Committee (No. S20/02/039) and MOI University, Institutional Research and Ethics Committee (No. IREC/2020/141) and National Commission for Science, Technology & Innovation (No. 957742) and before phase one of the study, permission was sought from the referral hospital itself. A waiver of consent was sought during the request for approval by the referral hospital. The researcher provided a clear anonymisation and data aggregation plan with the waiver application.

The study participants were provided adequate verbal and written information on the research before the study. An information leaflet was provided to the participants, and information on its contents was understood. The participants were informed that the research was voluntary, and they could withdraw at any stage of the study.

The participant’s interest was protected through respect for privacy and from undue pressure during data collection. No names were used in the transcripts to identify the participant, and the names on the consent form were coded so that the transcript only had a participant’s code.

All data are being kept under lock and key for the next 5 years and audio recordings are held in at the relevant department to which the first author is affiliated with, under the stipulated policy on storage of data.

## Results

### Synthesis and interpretation

***Quantitative:*** Maternal near-miss ratio (MNMR) was 8.7 per 1000 live births. The most prevalent direct factors were: blood transfusion (79%), severe postpartum haemorrhage (35%), eclampsia (18.9%), severe pre-eclampsia (17.4%) and hepatic dysfunction (3.7%). The most important contributory factors were anaemia, previous caesarean section and prolonged/obstructed labour. The prevalence of organ dysfunction at admission was 39%. Only 74% of eclampsia cases had received magnesium sulphate on referral. Higher gestation at delivery (AOR = 0.640, 95% CI = 0.477–0.858) and those who received antenatal care from a level 2 or 3 facilities (AOR = 0.190, 95% CI = 0.042–0.856) were less likely to experience a near miss. The authors found a higher burden of near misses among facilities located in the eastern region than in the western drainage.

Continuity of care index (COCI) and modified continuity of care index (MCCI) were lower among near-miss survivors compared to normal deliveries (COCI = 0.80, *p* = 0.0026), (MCCI = 0.62, *p* = 0.034). Near-miss cases scored lower on items assessing coordination between a higher-level provider and usual antenatal clinic (mean = 3.6, *p* = 0.006) and general care coordination during pregnancy (mean = 3.9, *p* = 0.019). In adjusted analysis, at least one non-life-threatening morbidity in pregnancy was associated with a near miss (aOR = 4.34, *p* = 0.001).

***Qualitative:*** The authors *deductively* identified three themes. The most discussed theme was collaboration of healthcare providers and health teams across facilities, and the authors labelled this *sequential coordination.* The second theme related to relationships between healthcare teams and women across the continuum of care and the authors marked this *continuity.* The third theme was collaboration between healthcare professionals and teams in primary healthcare settings, and the authors called this *parallel coordination.* Finally, some factors did not fall into the categories of continuity and coordination but contributed to the overall outcomes. These were classified as *access.*

In our analysis, woman-centred care underpinned *interpersonal continuity*, including sensitivity to the woman’s needs and avoiding stigmatising language. Accommodation through special arrangements such as special hotlines and community follow-up enhanced longitudinal continuity by keeping the woman in constant touch with health providers. *Sequential coordination* was related to collaboration between referring and referral facilities and power dynamics between providers at the two levels. *Parallel coordination* was influenced by optimal task shifting within facilities, management of workloads among nurses and midwives, and involvement of community health volunteers as part of the primary healthcare team. Finally, *access* factors included empowerment of women in reproductive health, family support, timely screening and identification of high-risk mothers and cost of care in referral facilities.

### Integrated findings

The authors conducted mixed methods integration based on qualitative and quantitative results to answer specific research questions. The questions addressed were: (1) how qualitative findings explain low continuity of care indices among near-miss survivors compared to non-near-miss cases, (2) why women who had at least one non-life-threatening morbidity in pregnancy are more likely to experience a near miss and (3) why there is a difference in the burden of near miss in eastern versus western regions. Table 3 presents a joint display of the mixed methods integration, where the meta-inference identifies whether qualitative findings explained, corroborated or enhanced the quantitative results.

**TABLE 3:** Joint display of integrated findings.

Quantitative findings	Statistical measures	Qualitative findings	Deductively identified qualitative themes	Illustrative quote	Meta-inference
Near miss survivors were less likely to visit the same facility during antenatal care (↓ longitudinal continuity)	Continuity of care index (↓ among near misses) Modified continuity of care index (↓ among near misses) Likert scale item scores	Subjective feelings of being cared for Interpersonal relationships	Longitudinal and relational continuity	*‘For that facility, you lie on the bed, immediately they place that thing for listening, after they listen, they tell you that you are ok, please climb down. You see, maybe you have come with some issues, the way am telling you I was in so much pain and my legs, I wasn’t able to walk well. So, you want them to touch you, so that you know why the pain is there. So they didn’t do that … so I went to facility (X)* …’ (woman 05)	Qualitative findings *explain* the association between relational and longitudinal continuity. *Woman-centred care* underpinned by good interpersonal relationships seems to foster longitudinal continuity. Other considerations include seeing the same team of healthcare providers and promoting accommodation strategies.
Near-miss survivors reported that their care was not well coordinated across facilities (sequential coordination)	Likert scale scores	Near miss experiences attributed to lack of collaboration between facilities leading to delayed care.	Sequential coordination	*‘…. In fact, the people at [facility X] really blamed [facility Y], saying that you should have brought this patient earlier, why did you wait until she worsened for you to make a phone call? You should have called early. Although when they realized things are tough, they switched off the phone, they “no longer received phone calls …”’* (woman 05)	Qualitative findings *confirm* perceived poor coordination of services among near-miss survivors and provide a potential explanation, i.e. the tension between HCPs in facilities. Therefore, teamwork emerges as an area of focus, highlighting the need for streamlining primary and specialist care referral pathways and processes.
Women who had at least one non-life-threatening morbidity in pregnancy were more likely to experience a near miss.	Odds ratios in multivariable logistic regression	Women with morbidities such as pre-eclampsia did not follow up on specialist consultation but ended up with complications at term.	Potentially parallel coordination but also an aspect of access.	*‘Actually most of the clients even the pre-eclampsia clients and the pregnancy-induced clients, most of them we usually advise them to go to* [*RH-B*], *even the multiple pregnancies, even the breech but you find the situation whereby you are at night, the same client who, even if you check on the ANC book, the same client was referred to the high-risk clinic, but the same same client comes during labour, with complications*’ (midwife 01)	This phenomenon may be related to both parallel coordination but also access issues. This highlights a need for innovative approaches such as collaboration between interdisciplinary teams, Care coordination roles (e.g. case and care managers, system navigators), individualised and tailored care plans, self-management support, specialist support and training.
The difference in the burden of near misses found between facilities	Prevalence of near miss in %	Constraints in human resources, laboratory test kits and ambulance services were more pronounced in the high near-miss facility. (*Observation*)	Access and/or human and physical resources	*‘… we have a good referral system although we don’t have vehicle here, we have one at Moi’s bridge health centre most of the time when we call them we are able to reach the ambulance and if it’s not available, we have a call centre at UGDH where they look for an ambulance from anywhere, any corner so long as they send an ambulance to us’* (midwife 04)	Qualitative findings potentially *explain* the difference in the burden of near misses between the two referral regions. Disparities in resources between the two regions may explain the relatively high near-miss burden in the western region compared to the other areas.

HCP, health care providers.

## Discussion

This study reinforces our knowledge of the complex health system factors that lead to avoidable near misses (and mortality) common in settings such as Kenya. Although the issues arising align with other quantitative and qualitative studies in this area (Akrawi et al. 2017; Assarag et al. 2015; Echoka et al. 2014; Lazzerini et al. 2018; Liyew et al. 2018; Yemane & Tiruneh 2020) mixed integration in this study offers unique insights.

### Longitudinal and interpersonal continuity

In this study, mixed-methods integration explained why near-miss cases had low COC. This is through near-miss survivors’ accounts of their experience during antenatal visitation. Mothers are likely to be more satisfied when they consult the same team of providers across pregnancy, labour and delivery (Sandall et al. 2016). This study underscored some mechanisms that promote this sense of satisfaction, namely the subjective feeling of being cared for, less stigmatisation of infertility or obstetric problems and general sensitivity from healthcare providers. Nevertheless, healthcare providers may not have adequate time to foster therapeutic relationships with mothers. There may be a need to balance the workloads and time for building provider–mother relationships in this setting. The amount of time spent may be less critical than the general respect for the woman. Respectful maternal care requires that healthcare providers treat the woman with respect, provide information, ask about her expectations and involve her in decisions about her care (Miller et al. 2016).

### Sequential coordination

Qualitative findings *confirmed* perceived poor coordination of services among near-miss survivors. The continuum of care in maternal healthcare is dependent on sequential coordination, where teamwork, professional leadership and communication are crucial determinants of positive outcomes (Cornthwaite, Alvarez & Siassakos 2015). However, our findings show a significant lack of teamwork in this critical area, pointing to the need to address this issue. The breakdown in collaboration could arise because of multiple factors, such as unannounced emergency referrals, a lack of adequate referral information and poor feedback (Echoka et al. 2014). In addition, hierarchies, cadre stereotyping and power dynamics could also play a role. Emergency Obstetric Care (EMOC) training was designed to promote teamwork during emergencies. However, the training approach primarily focuses on using simulation to mimic real-life emergencies, leaving little time devoted to non-technical aspects of collaboration, such as tolerance, willingness to accept divergent views and fostering collegiality. This approach may not be sufficient in promoting effective collaboration in real-life emergencies. Furthermore, quantifying the effect of poor collaboration on maternal outcomes is complex, making it challenging to emphasise its importance over other critical factors such as the lack of magnesium sulphate.

Recognising the need for cooperation and integration into EMOC training is the challenge that healthcare providers must face. Developing effective collaboration skills, including tolerance, communication and leadership, must be integrated into EMOC training to promote better outcomes for mothers and babies.

### Parallel coordination

Mixed integration highlighted the lack of collaboration between providers in the same setting as a potential driver of near-miss cases. Specifically, failure to follow up high-risk women identified in the first trimester was prominent. Community health volunteers are part of the workforce in primary health facilities and are tasked with providing preventive and promotive services at the household level, including follow-up care for high-risk women. Therefore, parallel coordination efforts should recognise and reinforce the work of community health workers as a link between health facilities and expectant mothers at home, particularly those identified as high risk. The effectiveness of community health workers’ work at the household level has been demonstrated in various areas, including eclampsia (Sevene et al. 2021), early Antenatal Care (ANC) attendance and adherence to iron and folic supplementation (Olaniran et al. 2019; Regan et al. 2022), among other interventions.

### Access

In this study, mixed-methods integration highlighted the problem of inequity in the distribution of physical and human resources as a determinant of obstetric near misses. Fair geographic distribution of resources is a fundamental measure of a given health system’s performance (Braveman & Gruskin 2003). The country where this study was done meets the WHO ratio of five EMOC facilities per 500 000 population, but such overall estimates may mask inequalities within and between regions. For example, a study in Malindi showed that 30% of facilities were misclassified as EMOC facilities because of a lack of critical human and physical resources (Echoka et al. 2013). This unequal distribution represents a severe risk of dying if a complication arises because the chances of getting to a comprehensive EMOC facility on time are diminished. This study also highlighted the limits of ‘improvisation’ in primary healthcare facilities. There is a need for policymakers to address fair distribution and access to EMOC services if the goal of reducing near-miss cases and mortality is to be realised.

Another critical access issue highlighted in mixed integration was the burden of out-of-pocket costs. This was cited as a reason why high-risk women identified at primary care centres do not follow up on referrals. The main driving factor for non-follow up appears to be out-of-pocket costs, as women are required to pay to access specialised services and diagnostics (Orangi et al. 2021). However, failure in coordination between primary healthcare facilities and households may also contribute to this problem.

### Limitations

The findings of the study may have limited generalisability because of its restriction to a single hospital setting. In addition, the retrospective chart review method used in the quantitative phase may have resulted in underestimation or overestimation of near-miss cases, as the documentation of obstetric care may have been incomplete or inaccurate. Moreover, the small sample size of the survey conducted in the quantitative phase (99 near-miss survivors and 112 normal deliveries) may have limited the statistical power of the analysis. The reliance on self-reported data from post-natal mothers in the quantitative phase may have introduced recall bias or social desirability bias. The purposive selection of participants in the qualitative phase, based on the quantitative phase’s results, may have introduced selection bias, potentially limiting the full range of experiences and perspectives related to obstetric near-miss cases. Finally, the study’s focus on only collecting data from near-miss survivors and healthcare providers may limit the understanding of other key stakeholders’ experiences, such as family members or community members.

## Recommendations for practice and research

### Longitudinal and relational continuity

More research is needed into how expectant mothers can be encouraged to consult the same midwife or group of midwives across pregnancy (and discourage them from changing facilities). Ongoing studies such as group antenatal visitation are essential to inform policy. However, solid evidence of these antenatal care models’ effect on mortality and morbidity outcomes must be substantial. Healthcare providers in primary healthcare facilities should be encouraged to design creative strategies to increase their engagement with expectant mothers and organise access to care, for example, through telehealth appointment systems and friendly patient flow systems.

### Sequential coordination

There is a need to promote situational awareness, teamwork and collaboration among nurses and midwives during emergencies, which may involve strengthening training and education on cooperation within and between levels of the healthcare systems. Specifically, there is a need to redesign midwifery training (basic, post-basic and continuing) towards more team-based approaches. Emergency Obstetric Care training should involve a more substantial component of collaboration and teamwork. Formal assessment tools for teamwork may be adopted into midwifery practice to entrench collaboration.

There may be a need to pilot novel care coordination roles in maternal health care (e.g. case and care managers, system navigators) to reduce fragmentation between levels of care. Approaches used in primary care and mental health may be adapted more to maternal health in resource-poor settings (WHO Secretariat 2016). System navigators for expectant mothers have been piloted in Australia (Baldwin et al. 2019). Such approaches will address the fragmentation of care, such as in our study, whereby women identified as high risk in the first trimester could not find specialist consultations. As a start, community health volunteers may double up as care navigators.

### Parallel coordination

Recognising and strengthening community-based follow-up as part of parallel coordination is critical. This may involve revisiting the role of Pearson correlation coefficient in the follow-up of high-risk women in the community – although this ultimately depends on the willingness of county governments (in Kenya) to incentivise and motivate lay health workers. Staffing norms should be applied equally across regions and facilities. Furthermore, the same attention given to task-shifting from physicians to nurses and/or midwives should be given to task-shifting from midwives to different cadres. Task-shifting to unqualified personnel should be monitored and discouraged as much as possible.

### Access

Novel and existing efforts at relieving the burden of out-of-pocket payments for specialist consultation, diagnostics and treatment should remain a priority. A recent study evaluating the free maternity programme in the country in which the study was done showed that mothers are still charged out-of-pocket fees, yet it is part of the benefits package (Orangi et al. 2021). Policy-level interventions for ensuring fidelity to the benefits package of the free maternity programme in Kenya are essential.

## Conclusion

The study examined the factors that contribute to poor continuity and coordination of care in maternal health in Kenya. Longitudinal and interpersonal continuity of care is crucial to mothers’ satisfaction, but healthcare providers may not have enough time to build therapeutic relationships. Poor teamwork and coordination of services also contribute to negative outcomes and training for collaboration and teamwork among healthcare providers is needed. Inequitable distribution of physical and human resources is another challenge to access to care, and policymakers should address this issue. The study recommends strategies to increase engagement between healthcare providers and expectant mothers and the use of care coordination roles and system navigators to reduce fragmentation between levels of care. It also suggests policy-level interventions to ensure fidelity to the benefits package of the free maternity programme in the country in which the study was carried out.
